# Human Precision-Cut Liver Slices: A Potential Platform to Study Alcohol-Related Liver Disease

**DOI:** 10.3390/ijms25010150

**Published:** 2023-12-21

**Authors:** Una Rastovic, Sergio Francesco Bozzano, Antonio Riva, Arturo Simoni-Nieves, Nicola Harris, Rosa Miquel, Carolin Lackner, Yoh Zen, Ane Zamalloa, Krishna Menon, Nigel Heaton, Shilpa Chokshi, Elena Palma

**Affiliations:** 1 The Roger Williams Institute of Hepatology, Foundation for Liver Research, London SE5 9NT, UK; 2 Faculty of Life Sciences and Medicine, King’s College London, London WC2R 2LS, UK; 3 Institute of Liver Studies, King’s College London, London WC2R 2LS, UK; 4 Institute of Pathology, Medical University of Graz, 8010 Graz, Austria

**Keywords:** ethanol hepatotoxicity, organotypic culture, ex vivo models, alcoholic hepatitis, fibrosis

## Abstract

Alcohol-related liver disease (ALD) encompasses a range of pathological conditions that are complex to study at the clinical and preclinical levels. Despite the global burden of ALD, there is a lack of effective treatments, and mortality is high. One of the reasons for the unsuccessful development of novel therapies is that experimental studies are hindered by the challenge of recapitulating this multifactorial disorder in vitro, including the contributions of hepatotoxicity, impaired lipid metabolism, fibrosis and inflammatory cytokine storm, which are critical drivers in the pathogenesis of ALD in patients and primary targets for drug development. Here, we present the unique characteristics of the culture of human precision-cut liver slices (PCLS) to replicate key disease processes in ALD. PCLS were prepared from human liver specimens and treated with ethanol alone or in combination with fatty acids and lipopolysaccharide (FA + LPS) for up to 5 days to induce hepatotoxic, inflammatory and fibrotic events associated with ALD. Alcohol insult induced hepatocyte death which was more pronounced with the addition of FA + LPS. This mixture showed a significant increase in the cytokines conventionally associated with the prototypical inflammatory response observed in severe ALD, and interestingly, alcohol alone exhibited a different effect. Profibrogenic activation was also observed in the slices and investigated in the context of slice preparation. These results support the versatility of this organotypic model to study different pathways involved in alcohol-induced liver damage and ALD progression and highlight the applicability of the PCLS for drug discovery, confirming their relevance as a bridge between preclinical and clinical studies.

## 1. Introduction

Alcohol-related liver disease (ALD) is one of the leading causes of high alcohol-attributable mortality worldwide and is responsible for 50% of global liver cirrhosis cases [1]. Despite this high incidence, ALD remains a clinically unmet need. The standard of care for ALD patients has not improved since the 1970s [2], and besides liver transplantation, there are no specific medications approved by the FDA for the treatment of this condition. Notably, the calculated attention-to-burden index for ALD is remarkably low compared to other liver diseases, demonstrating that research efforts invested in this disorder are inadequate [3].

ALD encompasses a complex spectrum of disease processes and, over sustained alcohol misuse, progresses through multiple stages from reversible histological manifestations, such as steatosis and inflammation, to most severe and deadly clinical conditions, including cirrhosis, hepatic decompensation and alcohol-associated hepatitis (AH) [4]. Key pathological features have been identified with relevant prognostic value [5], but molecular studies are required to advance our understanding of the disease mechanisms that can be therapeutically targeted. Multiple dysregulated molecular pathways synergistically contribute to the pathogenesis and progression of ALD through disease stages. Firstly, there is an impairment of lipid metabolism, while the toxic effect of alcohol and its detoxification directly affects the viability and functionality of the hepatocytes [4,6], where ethanol metabolism takes place. When the hepatic response to damage shifts from transient to persistent, such as in chronic alcohol misuse, all the mechanisms in place to maintain homeostasis tip the system into an abnormal steady state. With cycles of healing and scarring, there is excessive profibrogenic activation of the hepatic stellate cells in response to hepatocyte damage, inflammation, and altered deposition of collagen and other extracellular matrix (ECM) proteins, causing fibrosis and eventually cirrhosis. Additionally, chronic high alcohol consumption drives intestinal dysbiosis and altered gut permeability, leading to bacterial translocation from the gut driving further inflammation and immune dysfunction, key events in the late deterioration of ALD [4,7,8].

Given the multi-layered pathologic mechanisms active in ALD, successful treatment strategies should possess pleiotropic properties targeting different disease processes [2,9], which need assessment in appropriate experimental models to perform meaningful drug screening. For example, although many animal studies have been proven beneficial in investigating alcohol hepatotoxicity [10], they do not fully recapitulate the disease as seen in humans [11,12,13,14,15]. A relevant experimental model must recapitulate the multiple processes involved in the human disease (steatosis, hepatotoxicity, inflammation and fibrosis) and adequately represent patient heterogeneity to translate findings from bench to bedside or to “reverse translate”, that is, to mechanistically unpick the reasons for failed therapeutics in a relevant model.

Primary human hepatocytes have been used to study the effect of ethanol on different pathways [16,17,18], but in recent years, there have been advances in the use of human-derived experimental models of ALD such as liver-on-a-chip [19] and hepatic organoids [20]. In addition, hepatic organoids derived from liver tissue from patients with alcohol-associated hepatitis were used for the investigation of hepatocyte-to-biliary reprogramming in ALD [21], indicating the range of investigations that can be performed using patient-derived samples. Another organotypic model for the study of ALD is the ex vivo culture of precision-cut liver slices (PCLS), systematically produced using a special tissue microtome and utilised for a variety of applications [22,23]. A critical advantage of the liver slice model is that it enables the investigation of not only the impact of alcohol and its metabolism but also the contribution to disease progression driven by the specific cellular and non-cellular components of the liver in their native histoarchitectural organisation. PCLS have widely been recognised as a valuable tool for functional and toxicological studies [24,25,26] and disease modelling, with a prevalence of investigations related to fibrosis [27,28,29,30]. Only a few studies have focused on alcohol-related toxicity [31,32], mostly showing ethanol metabolism and associated oxidative stress or ethanol-induced steatosis, confirming the utility of the system for investigating these pathways, but this was limited to rat-derived slices [31,32,33]. Our group has previously shown the potential of human PCLS for studies on acute ethanol effects [7,34] and for the study of megamitochondria formation [35], which is one of the histological hallmarks of ALD [5]. Collectively, these results set the basis for a further investigation and characterisation of the critical processes associated with ALD development in the human slice culture to allow a deeper understanding of the processes involved in the disease and therefore translate results from preclinical research to clinical practice. The aim of this study was to explore in human PCLS the effects of extended alcohol exposure, steatosis, endotoxemia-related inflammation and fibrogenesis—all of which are critical components of the disease and important targets for therapeutic intervention.

## 2. Results

### 2.1. Human Precision-Cut Liver Slices Are Viable up to Six Days in Culture

**Figure 1:**
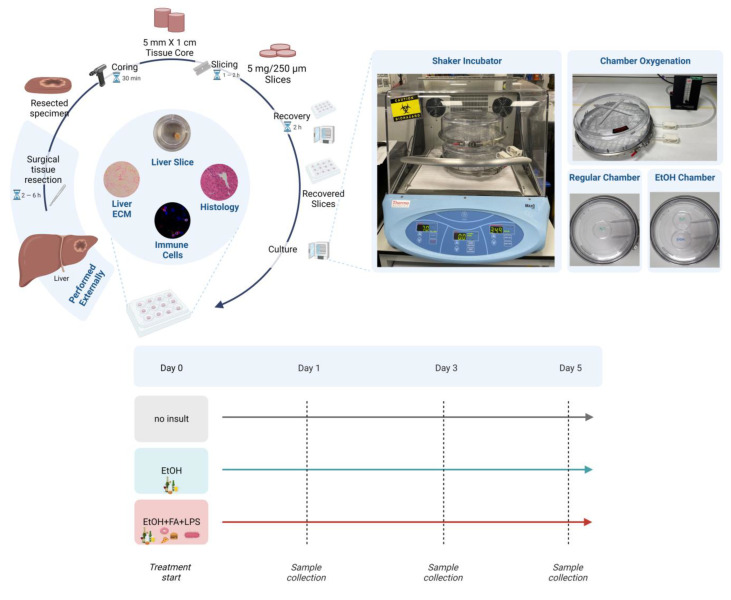
Human precision-cut liver slices (PCLS) preparation and culture. PCLS are prepared from tumour distal portion of resected liver tissue (referred to as baseline). Tissue cores are prepared with the coring press (5 mm in diameter, 1 cm long), and are sliced with the Krumdieck (Alabama R&D) tissue slicer to produce 5 mg PCLS (approx. 250 µm thick). After PCLS preparation, slices are cultured for 2 h during the recovery step. Slices are cultured in a shaker incubator, in carbogen chambers (95% O_2_, 5% CO_2_) containing a Petri dish with distilled water to prevent evaporation, incubated at 37 °C, and shaking at 70 rpm. After recovery, they are cultured overnight, and the treatment with alcohol insults starts the following morning (Day 0). Culture plates with slices treated with alcohol insults are kept separately in an EtOH chamber which in addition to distilled water contains a Petri dish with 500 mM ethanol to prevent ethanol evaporation from the medium. Figure created with BioRender.com.

**Figure 2:**
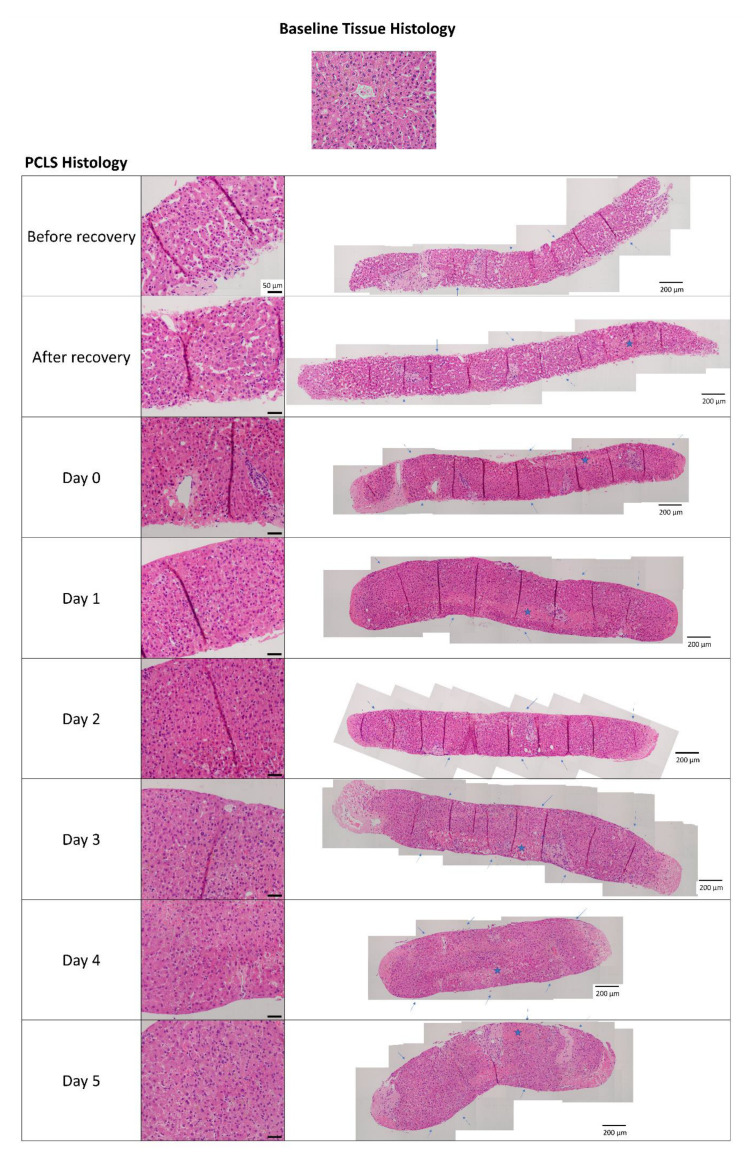
Representative H&E staining of human precision-cut liver slices (PCLS) over time compared to the baseline tissue used to obtain PCLS. Histology images show preserved liver histoarchitecture in PCLS and viable hepatocytes over time in culture. Middle panel: selected areas of the slice (scale bar: 50 µm), right panel: manually stitched image acquisition of the whole slice using Multiple Image Alignment (MIA) option in Olympus cellSens Standard 2.3 software (scale bar: 200 µm). Magnification: 400×. Blue arrows indicate the integrity of the tissue edge/cut surface over time; blue stars indicate cell damage, i.e., areas of necrosis, at or parallel to the cut surface of the slice. The pattern of cell damage did not follow lobular organisation.

A longitudinal assessment of slice histology and markers of viability or cell death (quantification of ATP content in the tissue, release of cytokeratin-18 in culture supernatants and PCLS weight) was performed to monitor the PCLS culture before setting up the ALD model as illustrated in Figure 1. The longitudinal viability assessment is essential when utilising human tissue, given the high clinical heterogeneity (Table 1). The histological and molecular analysis started immediately after slicing (referred to as “Before recovery” in Figure 2). As expected, and in line with previous reports [26,30,36], the mechanical cut during PCLS preparation induced some damage to the tissue, but the cell death and loss of tissue integrity were minimal when PCLS were compared to the histological specimen from the Pathology department (immediately fixed after surgery) (Figure 2).

**Figure 3:**
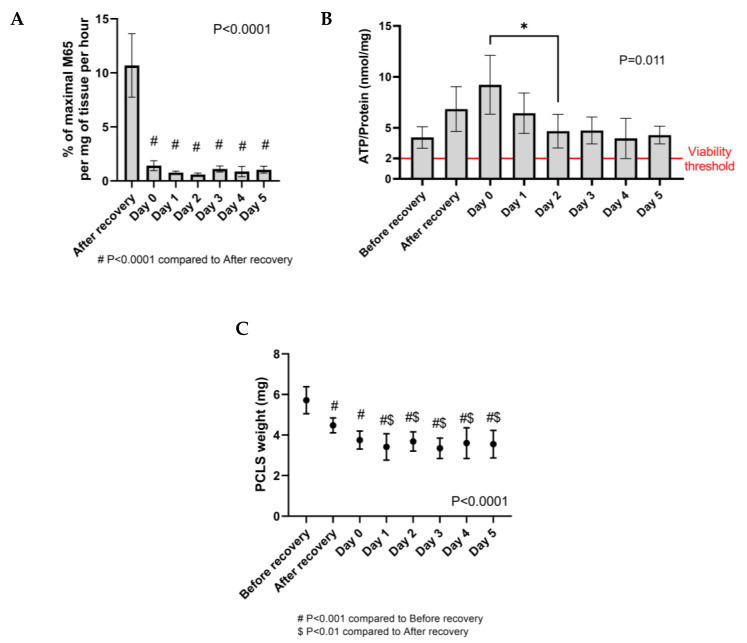
Longitudinal assessment of human precision-cut liver slices (PCLS) culture viability. (**A**) Hepatocyte death rate measured as a % of maximal M65 release per mg of tissue per hour in culture supernatants. Measurement of M65 in culture supernatants was only possible from the after-recovery timepoint onwards as this is the first timepoint when the slices are cultured. M65 release was measured after 2 h incubation at the after-recovery timepoint, after 15 h incubation at Day 0 and after 24 h incubation at timepoints Day 1–Day 5. Mean ± 95% CI. n (patients) = 7; minimum 3 samples per patient. (**B**) PCLS viability measured as ATP content in the tissue. Mean ± 95%CI. The full red line indicates the viability threshold (2 nmol/mg ATP/protein). n (patients) = 5, minimum 3 samples per patient. (**C**) PCLS weight over time. Mean ± 95% CI. n (patients) = 7, minimum 3 samples per patient. * p ≤ 0.05.

Moreover, before the addition of alcohol insults, PCLS underwent a “recovery step” to remove cells damaged during slice preparation, namely a short 2 h long dynamic culture at 37 °C in a carbogen atmosphere, followed by an overnight culture in the same conditions with the medium replaced at every step (see Section 4). The recovery step and the overnight culture facilitated the shedding of damaged cell layers and improved tissue integrity as seen from the well-defined tissue edges (Figure 2, blue arrows). This time significantly reduced the hepatocyte death rate, as seen by the substantial drop at Day 0 in the levels of cytokeratin-18 (M65), a marker of epithelial cell death (Figure 3A), and supported the increase in ATP tissue content (Figure 3B), indicative of the re-activation of metabolic processes after cold storage. This increase in ATP tissue content following PCLS preparation is in line with observations from our previous study [35].

Additionally, the histology and viability of the slices were investigated at every critical step of the culture, namely the daily change of medium, until the last time point (Day 5). Liver histoarchitecture was preserved in the PCLS, and the hepatocytes were viable, as seen from the H&E staining (Figure 2). Moreover, the ATP per protein content in the PCLS was above the viability threshold (2 nmol ATP/mg protein [26]) at all timepoints. Importantly, the ATP/protein levels remained stable from Day 1 onwards (Figure 3B), which is relevant for our study on the impact of alcohol insults. Finally, the cell death levels were very low and stable along the Day 1–5 culture (Figure 3A), as was the slice weight (Figure 3C). Taken together, given the high ATP content and low levels of cell death, Day 0 was selected as the appropriate time to start alcohol exposure, which also facilitated the logistics of longitudinal collection at Days 1, 3 and 5. Also, cultures that were not viable for the whole duration of the experiment were excluded from further analysis (Supplementary Figure S1).

### 2.2. Upregulation of Lipid Synthesis in Human Precision-Cut Liver Slices Exposed to Alcohol

**Figure 4:**
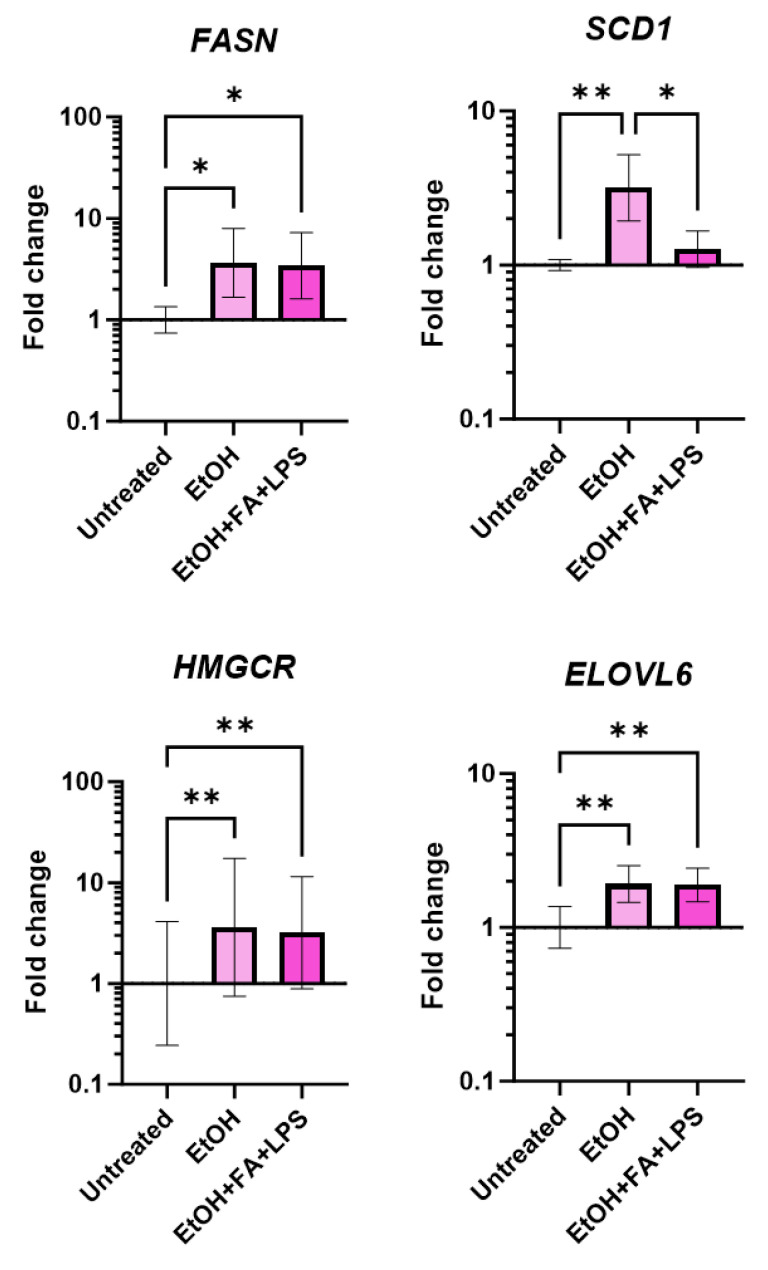
Expression of genes involved in lipid synthesis and remodelling in human precision-cut liver slices (PCLS) exposed to alcohol insults. Change in gene expression in the PCLS induced by the insults quantified in PCLS cultured for 3 days, normalised per no-insult control. Geomean ± SD. * *p* ≤ 0.05, ** *p* ≤ 0.01; n (patients) = 3, 1 sample per patient.

The impact of ethanol alone (EtOH) or in addition to fatty acids (FAs) and LPS (to mimic the bacterial translocation seen in patients) was investigated at Days 1, 3 and 5 in the slice cultures confirmed viable via the longitudinal assessment previously described. One of the early and most common manifestations of ALD is the development of steatosis or fatty liver. Alcohol causes the accumulation of lipids in hepatocytes through the inhibition of beta-oxidation and the promotion of de novo lipogenesis through several molecular mechanisms [37]. These changes in PCLS treated with alcohol insults were assessed by gene expression of key enzymes involved in de novo lipogenesis (*FASN*, *SCD1*) [37], cholesterol synthesis (*HMGCR*) [38] and fatty acid remodelling (*ELOVL6*) [39]. PCLS ex vivo recapitulate the imbalance in lipid synthesis associated with ALD, as seen from the upregulation of gene expression of *FASN*, *SCD1*, *HMGCR* and *ELOVL6* following treatment with ethanol alone compared to the untreated control (Figure 4). The same effect was observed with EtOH + FAs + LPS, but no significant changes were detected in the expression of SCD1 compared to the untreated slices.

### 2.3. Alcohol Insults Induce Hepatocyte Damage in Human Precision-Cut Liver Slices

**Figure 5:**
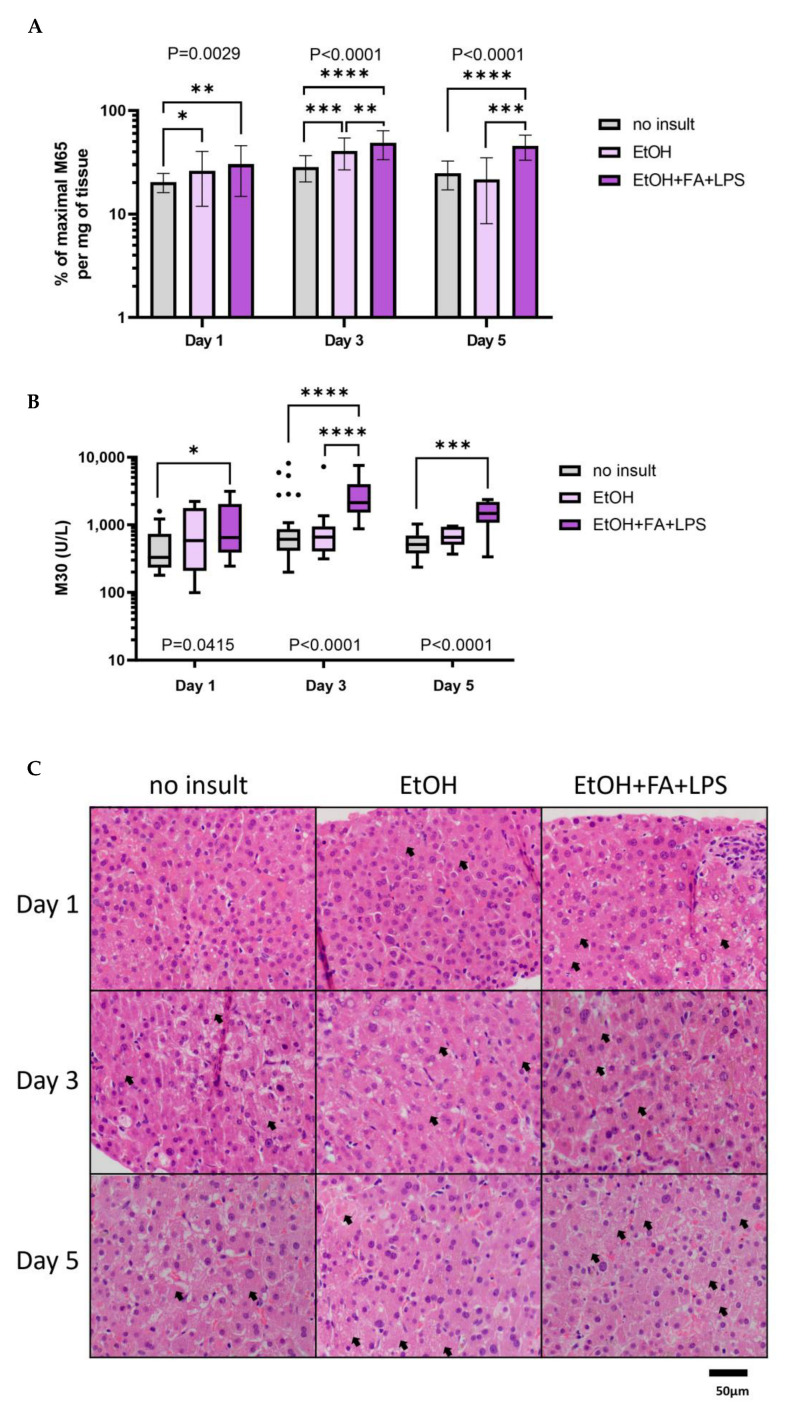
Hepatotoxic effect of alcohol insults on human precision-cut liver slices (PCLS). (**A**) Total hepatocyte death measured as a release of M65 epitope of cytokeratin-18 in culture supernatants expressed as a percentage of maximal M65 release per mg of tissue. Geomean ± 95% CI. * *p* ≤ 0.05, ** *p* ≤ 0.01, *** *p* ≤ 0.001, **** *p* ≤ 0.0001; n (patients) = 3–7 per timepoint, minimum 3 samples per patient. (**B**) Apoptosis observed in PCLS measured as a release of M30 epitope of caspase-cleaved cytokeratin-18 in culture supernatants. Tukey box plot. * *p* ≤ 0.05, ** *p* ≤ 0.01, *** *p* ≤ 0.001, **** *p* ≤ 0.0001; n (patients) = 3–7 per timepoint, minimum 3 samples. (**C**) Representative H&E staining of PCLS treated with alcohol insults over time. Black arrows: hepatocyte damage (swelling of cells, pale cytoplasm, loss of nuclei). Scale bar: 50 µm, magnification: 400×.

Alcohol-associated epithelial cell death in PCLS, predominantly coming from dying hepatocytes, was measured by the release of the M65 epitope of cytokeratin-18 (total death) and the caspase-cleaved M30 epitope of cytokeratin-18 (apoptosis) in culture supernatants, selected as a clinically relevant marker and associated with severe hepatic inflammatory activity [40,41,42]. Both alcohol insults tested (EtOH and EtOH + FAs + LPS) induced significant total cell death, as evident from the release of the M65 epitope (Figure 5A). EtOH treatment increased the mean percentage of weight-adjusted M65 release after 24 h of exposure when compared to the respective untreated control, as seen both at Day 1 (*p* = 0.0438) and at Day 3 (*p* = 0.0005). The combination of EtOH, FAs and LPS increased M65 epitope release compared to the untreated control at Day 1 (*p* = 0.0036), Day 3 (*p* < 0.0001) and Day 5 (*p* < 0.0001). Moreover, EtOH, FAs and LPS induced more cell death compared to ethanol alone, as seen from the significant increase in M65 release at Day 3 (*p* = 0.0050) and Day 5 (*p* = 0.0001) (Figure 5A).

The modality of cell death also seemed different between ethanol alone and with the addition of FAs and LPS as only the latter induced a significant increase in apoptosis in PCLS (Figure 5B). The mean concentration of the M30 epitope of cytokeratin-18 was significantly increased at Day 1 (*p* = 0.0493), at Day 3 (*p* < 0.0001) and at Day 5 (*p* < 0.0001) with EtOH, FA and LPS treatment compared to the respective untreated controls. These results were also confirmed with TUNEL staining and quantification of apoptotic cells (Supplementary Figure S2).

PCLS histology in the presence of alcohol insults was assessed by H&E staining. Hepatocyte damage was more pronounced in PCLS treated with insults compared to untreated control (Figure 5C, black arrows showing cell swelling, pale cytoplasm and loss of nuclei). Additionally, there was no difference in weight change between PCLS treated with alcohol insults and the control (Supplementary Figure S3).

### 2.4. Differential Inflammatory Responses Are Triggered by EtOH Alone or in Combination with FA and LPS Treatment in Human Precision-Cut Liver Slices

**Figure 6:**
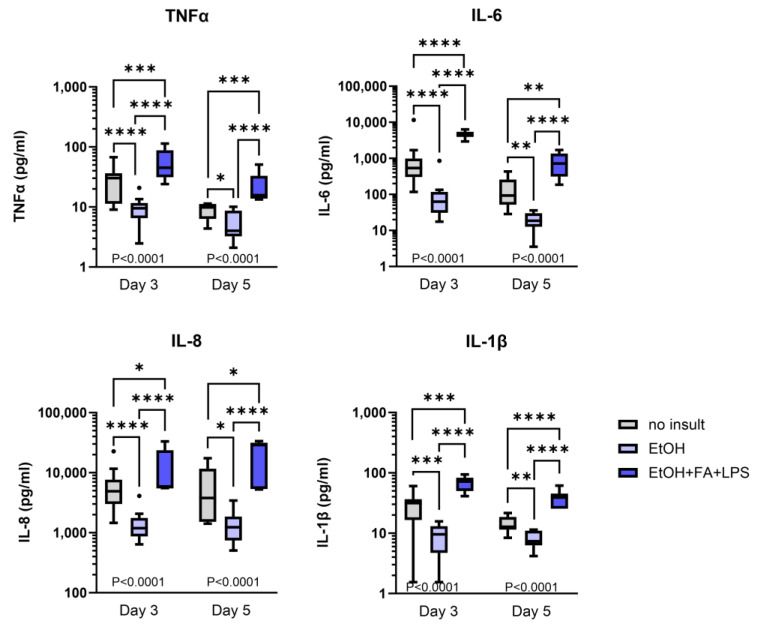
Release of proinflammatory cytokines in human precision-cut liver slices culture supernatants following alcohol treatment. Tukey box plot. * *p* ≤ 0.05, ** *p* ≤ 0.01, *** *p* ≤ 0.001, **** *p* ≤ 0.0001; n (patients) = 3–6 per timepoint, 1–3 samples per patient.

The development of inflammation in human slices exposed to alcohol stimuli was measured by the release of four innate proinflammatory cytokines in the culture supernatant: TNFα, IL-6, IL-8 and IL-1β. PCLS maintained the ability to respond to proinflammatory stimuli and showed a significant increase in the release of all cytokines measured (TNFα, IL-6, IL-8, IL-1β) at both Day 3 and Day 5 with EtOH, FA and LPS treatment (Figure 6). On the contrary, the single treatment with EtOH induced a very consistent but opposing response showing significantly lower levels of all four cytokines compared to both the untreated control and the EtOH treatment including FAs and LPS, at both timepoints Day 3 and Day 5 (Figure 6).

### 2.5. Fibrogenic Activity Was Observed in Human Precision-Cut Liver Slices Independently from Alcohol Stimuli

**Figure 7:**
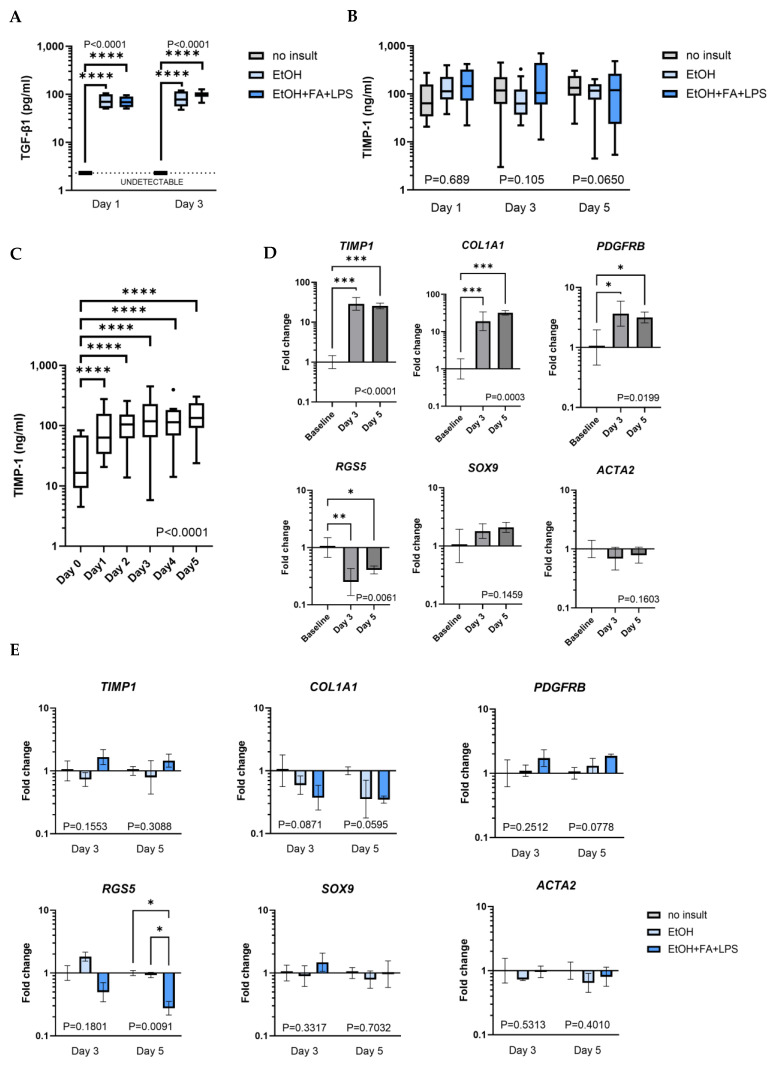
Profibrotic changes in human precision-cut liver slices (PCLS). (**A**) Release of the active form of TGF-β1 in culture supernatants. Tukey box plot. **** *p* ≤ 0.0001; n (patients) = 2–3 per timepoint, 2 samples per patient. (**B**) TIMP-1 release in culture supernatant in the presence of insults. Tukey box plot. n (patients) = 3–7 per timepoint, minimum 3 samples per patient. (**C**) TIMP-1 release in culture supernatant over time. Tukey box plot. **** *p* ≤ 0.0001; n (patients) = 3–7 per timepoint, minimum 3 samples per patient. (**D**) Change in gene expression induced by the cut during PCLS preparation quantified in PCLS cultured for 3 and 5 days, normalised per baseline uncut tissue that was used to obtain PCLS (Figure 1). Geomean ± SD. * *p* ≤ 0.05, ** *p* ≤ 0.01, *** *p* ≤ 0.001; n (patients) = 5, 1 sample per patient. (**E**) Change in gene expression in the PCLS induced by the insults quantified in PCLS cultured for 3 and 5 days, normalised per no-insult control for each timepoint. Geomean ± SD. * *p* ≤ 0.05; n (patients) = 5, 1 sample per patient.

The fibrotic response to alcohol was initially evaluated in human PCLS by the quantification of fibrogenic markers (TIMP-1 and TGF-β1) in culture supernatants at Days 1, 3 and 5 (Figure 7A,B). The release of TGF-β1 stood out as a significant effect induced by alcohol insults, while the untreated controls had undetectable levels of active TGF-β1 (Figure 7A). Remarkably, the levels of TIMP-1 detected in slices unexposed to insults were comparable to samples treated with alcohol insults (Figure 7B). This led to an additional longitudinal analysis performed in the absence of alcohol insults, which showed a significant increase in released TIMP-1 over time (Figure 7C). The mean concentration of TIMP-1 at Day 1 (mean log_10_ = 1.854) and Day 2 (mean log_10_ = 1.956) was significantly higher than that at Day 0 (mean log_10_ = 1.322), corresponding to geomean fold changes of 3.30 (*p* < 0.0001) and 4.87 (*p* < 0.0001) from Day 0 to Day 1 and Day 2, respectively. After this initial increase, the mean TIMP-1 release did not change significantly from Day 1 onwards. These data suggested the activation of fibrogenic processes in culture independently from the addition of alcohol treatment, and the expression of fibrogenic markers was therefore evaluated in PCLS cultured for 3 or 5 days and this time compared to the baseline expression in the tissue of origin, snap-frozen at the time of PCLS preparation but before slicing (Figure 7D). The significant upregulation in the slices of *TIMP1*, *COL1A1* and *PDGFRB* expression demonstrated a strong effect associated with slicing, with a 28.12-fold mean increase in *TIMP1* (*p* < 0.0001), a 17.78-fold mean increase in *COL1A1* (*p* = 0.0003) and a 3.48-fold mean increase in *PDGFRB* (*p* = 0.0199) gene expression at Day 3. The expression of *RGS5*, whose downregulation has been recently reported following liver injury [43], was significantly downregulated in the PCLS compared to baseline, with a mean 0.25-fold decrease (*p* = 0.0061) at Day 3. The expression of *SOX9* and *ACTA2* did not significantly change in the PCLS compared to the baseline tissue.

Considering the remarkable upregulation of the gene expression of fibrotic markers in untreated slices compared to baseline, alcohol insults did not have an additional effect on most genes. The only exception was *RGS5*, as its expression in PCLS treated with EtOH, FAs and LPS was significantly decreased compared to the untreated control (*p* = 0.0118) and slices treated with ethanol only (*p* = 0.0145) at Day 5 (Figure 6E). Notably, the addition of alcohol insults showed a trend of downregulation of *COL1A1* (*p* = n.s.), which was previously reported in rat slices [33].

## 3. Discussion

Several studies have highlighted the challenges in meaningfully replicating the multifactorial processes associated with ALD in experimental models [13,14,15]. The current work reveals the suitability of human liver slices for disease modelling for ALD to bridge this gap and focuses on recapitulating some of the main drivers of disease, namely steatosis, hepatotoxicity, inflammation and fibrogenesis. Moreover, this study highlights the essential requirement of a thorough characterisation of patient-derived multicellular models tailored around the specific project aims and pathways of interest. Successful therapies for ALD will require multipronged approaches to target different pathogenic mechanisms involved in the disease [2,6,9]. In this context, human PCLS constitute a valid option for preclinical studies, as multiple pathogenic mechanisms can be activated in tandem in a model that is species-relevant and maintains hepatocytes and non-parenchymal cellular compartments in their native environment ex vivo (Supplementary Figure S4) [23].

Here we showed that the PCLS are viable in culture, retain liver-specific histoarchitecture, show an imbalance in lipid synthesis following ethanol exposure, and respond to pro-inflammatory and hepatotoxic stimuli ex vivo. Firstly, we performed a thorough molecular and histological characterisation of the slice culture and ensured a relevant and appropriate assessment of the effects of two types of alcohol insults over a five-day treatment in order to model the alcohol-specific hepatic impact and other features associated with ALD. PCLS were exposed to higher levels of ethanol and LPS, and for a longer time compared to other studies on human slices [44,45]. The dose selection was based on our previous findings that, in this condition, PCLS develop hepatocyte megamitochondria [35], a peculiar marker of ALD and a phenomenon with clinical relevance. Additionally, although ethanol 250 mM may seem an excessively high dose, it is worth considering that in a recent study, Meijnikman et al. reported that the ethanol concentration in the portal vein blood could be up to approximately 500 times (interquartile range, 17–516) higher compared to that in peripheral blood [46]; therefore, the liver is indeed exposed to a very high dose. Interestingly, in the same study, the gut microbial production of ethanol was quantified in the portal vein blood of non-drinking patients (ethanol consumption of more than two units was an exclusion criterion) with MASLD (metabolic dysfunction associated steatotic liver disease) and compared with healthy individuals without hepatic steatosis (but overweight). Strikingly, the median ethanol concentration in portal vein blood in individuals with steatosis or steatohepatitis ranged from 8 mM to 21 mM, suggesting that in patients with chronic liver disease, the liver is already chronically exposed to ethanol (doses are higher than driving limits) regardless of alcohol consumption [46].

For the second type of insult, we combined ethanol with a mixture of fatty acids (oleic and linoleic acid, 0.1 mM) and 10 μg/mL LPS to induce the steatotic disease phenotype and pathological endotoxemia caused by bacterial translocation in ALD patients. Alcohol exposure promoted an increase in lipid synthesis and remodelling in PCLS. Interestingly, the gene expression of *SCD1*, steryl-CoA-desaturase-1 which converts saturated fatty acids to monounsaturated, was upregulated in PCLS treated with ethanol only but not in combination with FAs and LPS. This could be explained by the presence of monounsaturated oleic acid in the FA mixture that acts as a negative regulator for fatty acid desaturation. In addition, histology and M65 (epitope of cytokeratin-18) release in the culture supernatant confirmed that both types of alcohol insults induced hepatocyte death as early as after 24 h of exposure. Strikingly, the addition of fatty acids and LPS to ethanol significantly increased total cell death and apoptosis compared to ethanol alone, replicating what happens in vivo, where the contribution to hepatocyte damage comes from multiple hits and not only from the direct hepatotoxic effect associated with ethanol and its metabolism.

Indeed, the PCLS treated with EtOH, FAs and LPS showed an increase in the release of prototypical proinflammatory cytokines (TNFα, IL-6, IL-8 and IL-1β) resembling the hyperinflammatory state in patients. On the contrary, EtOH alone consistently downregulated the production of proinflammatory cytokines compared to the untreated control. More focused studies, i.e., targeting the immune compartment retained or resident in the slices, are required to determine the specific molecular mechanisms behind these differences.

Additionally, the PCLS showed profibrogenic activity, as seen by the analysis of gene expression and soluble markers. Notably, the preparation of the PCLS induces a wound-healing profibrogenic activity in response to the mechanical cut, which has been reported in slices before [30,33,36,47], and this was observed regardless of ethanol exposure. Despite having shared pathways, alcohol-related fibrogenesis represents a different type of response compared to the one induced by slicing, such as that seen in partial hepatectomy [48,49,50]. This is demonstrated in our results, as we show the detection of active TGF-β1 exclusively in the supernatants from cultures exposed to alcohol and not in the untreated controls. Interestingly, the evaluation of collagen staining with picrosirius red did not show consistent macroscopic differences following alcohol treatment (Supplementary Figure S5). Instead, this analysis highlighted a high risk of sampling bias when attempting to observe these changes histologically, as PCLS may contain areas naturally rich in connective tissue, such as portal triads, or areas with pre-existing fibrotic activation due to anticancer treatment of the tissue donor. This observation confirmed that the preferred way of assessing the changes in the slices is by using molecular techniques and that for assessing histological changes, more sensitive methods such as digital pathology could be used for the analysis of ECM fibre features [51,52].

In addition, we saw a significant increase in PCLS thickness over time, suggesting that structural changes are induced as a wound-healing response to the mechanical cut (Supplementary Figure S6). Remarkably, alcohol exposure had a substantial impact on the slice thickness, showing a significantly lower increase over the culture period. We can speculate that this may be related to an impairment in the wound-healing response in PCLS treated with ethanol, and this may be linked to a limitation of hepatocyte growth, as alcohol is a known inhibitor of hepatocyte proliferation [53,54,55]. Altogether, these findings suggest the important effect of alcohol on the regenerative capacity of the liver that is relevant for ALD development and is recapitulated in the PCLS.

A critical advantage of the model we propose in this study is the use of human specimens to model ALD. Previously, rodents have been mostly utilised for this purpose [31,32,33], as the tissue is easily accessible, the logistics straightforward and the issue of inter-individual variability less prominent. However, critical species-specific differences need to be considered, and an increasing number of publications highlight how rodent models do not reflect human diseases [11,13,14,15]. The discordance between humans and rodents is particularly relevant for alcohol-related investigations, as mice and rats are significantly more resistant to alcohol toxicity and differ in immunological and disease processes [12,56]. The use of patient-derived models would make it possible to reduce the gap between pre-clinical or clinical research and clinical practice. Moreover, the relevance of this work is further supported by the recent approval of the FDA Modernization Act 2.0, allowing alternatives to animal testing for investigating the safety and effectiveness of drugs [57,58].

However, the biggest limitation of the study also relates to the source of tissue. Although human models can more closely resemble disease processes as seen in patients, the heterogeneity of the tissue donors needs to be noted, and a robust assessment of culture viability and variability needs to be applied for each experiment. Nevertheless, it is encouraging to see that despite patient heterogeneity, the effects of alcohol insults in the slices are in line with the current understanding of molecular drivers of ALD [4,6]. Furthermore, as the use of biopsy in early disease is not approved, some of the processes in the pathogenesis of ALD are still poorly understood, and the human PCLS-ALD model is an excellent way to study and understand alcohol-induced damage in diverse subjects, which can ultimately help guide the clinical management of different ALD patient cohorts. Finally, tissue donor variability inevitably affects the uniformity of the results, increasing the need for multiple replicates from a greater number of subjects, but on the other side, it can translate into a closer representation of real-life studies. Moreover, for the investigation of the late stages of ALD, PCLS can be sourced from explants derived from patients with alcohol-related cirrhosis undergoing liver transplantation, broadening the range of pathological mechanisms that can be studied with this versatile and relevant model.

Taken together, these results support the suitability of the PCLS model for studying multiple molecular pathways involved in the pathogenesis of ALD. Moreover, this study paves the way for more targeted investigations as further research is needed to thoroughly elucidate the efficacy and additional uses of this method. Finally, we demonstrate that PCLS represent a potential platform for testing therapeutic regimens, including approaches synergistically targeting inflammation, hepatotoxicity, steatosis, fibrosis, the extracellular matrix or hepatocyte proliferation, possibly in combination with immunomodulators, increasing the probability of successfully treating different disease stages across the ALD spectrum [9,59].

## 4. Materials and Methods

### 4.1. Patient Recruitment

Human precision-cut liver slices were prepared from the tumour-free distal portion of liver tissue (as defined by the liver histopathologist) resected for the removal of colorectal liver metastases. Written informed consent was obtained from all subjects involved in this study. This study was conducted in accordance with the Declaration of Helsinki and approved by the local Research Ethics Committee established by the Health Research Authority (REC reference 17/NE/0340; IRAS project ID 222302). Tumour distal liver tissue was considered healthy; i.e., tissue donors did not have chronic liver disease. Tissue from seven patients was used for the PCLS preparation, and baseline clinical characteristics are summarised in Table 1.

### 4.2. Human Precision-Cut Liver Slices Preparation and Culturing Conditions

Collected human liver tissue was kept in sterile University of Wisconsin solution (ViaSpan; Bridge to Life Ltd., London, UK) until slicing. The PCLS (approximately 5 mg in weight, 250 µm thick) were prepared using a Krumdieck tissue slicer (Alabama R&D, Munford, AL, USA) as described previously [26,60]. The time from tissue collection to culture was kept to a minimum (approx. 3 h). Following slicing, the PCLS were cultured for 2 h (recovery step) and subsequently moved to fresh media until the following morning, which is referred to as the start of the experiment (timepoint: Day 0). The overall ex vivo culture length was 6 days in total (Day 0–Day 5).

One slice per well was cultured in a 12-well plate (Nunc, ThermoFisher Scientific, Oxford, UK) in 1.5 mL William’s E Medium (ThermoFisher Scientific, Oxford, UK) supplemented with 5% Human AB serum (Pan-Biotech, Wimborne, UK), Penicillin/Streptomycin (ThermoFisher Scientific, Oxford, UK), 2 mM Glutamine (ThermoFisher Scientific, Oxford, UK), ITS (10 mg/L Insulin + 5.5 mg/L Transferrin + 6.7 µg/L Sodium selenite, ThermoFisher Scientific, Oxford, UK), 1 nM epidermal growth factor (ThermoFisher Scientific, Oxford, UK), 100 nM Glucagon (Merck, Gillingham, UK) and 1 µM Corticosterone (Merck, Gillingham, UK). In order to maintain sufficient oxygenation of the middle cell layers, the PCLS were kept in high-oxygen conditions (95% O_2_, 5% CO_2_, BOC, Guildford, UK) in humidified carbogen chambers at 37 °C, gently shaking at 70 RPM in an orbital shaker incubator (ThermoFisher Scientific, Oxford, UK) creating a dynamic culture. The carbogen was refilled twice a day and after every opening of the chamber. The medium was replaced daily.

Hepatotoxicity was induced in the PCLS with two types of alcohol insults: ethanol (EtOH, 250 mM, Merck, Gillingham, UK) alone or in combination with free fatty acids (FAs, 0.1 mM combination of oleic and linoleic acid, Merck, Gillingham, UK) and lipopolysaccharide (LPS 10 µg/mL, Merck, Gillingham, UK). To prevent evaporation of the ethanol from the medium, plates with slices treated with ethanol were kept in a separate carbogen chamber (Billups-Rothenberg Inc., San Diego, CA, USA) which, in addition to a Petri dish with water, also contained an open Petri dish with 500 mM ethanol. The samples were collected for analysis after 24 (Day 1), 72 (Day 3) and 120 (Day 5) hours of exposure to alcohol insults (Figure 1). In addition to this, for all experiments, control slices were cultured in parallel, and the samples were collected at every medium change to monitor the PCLS in culture.

### 4.3. Histology

PCLS were fixed in 10% neutral buffered formalin (Fisher Scientific, Loughborough, UK) overnight at 4 °C. The fixed slices were dehydrated with ethanol washes, cleared with xylene (Genta Medical, York, UK) and embedded in paraffin. The paraffin blocks with the slices were cut with a microtome at 4 μm. The slides with the tissue were deparaffinised with xylene, rehydrated in ethanol baths and stained with Harris haematoxylin (Leica Microsystems, Milton Keynes, UK) and eosin (VWR, Lutterworth, UK) (H&E staining).

### 4.4. Quantification of the ATP Content in the PCLS

Tissue slices used for the measurement of the ATP content were snap-frozen in a 70% ethanol solution (2 mM EDTA, pH 10.9) and were kept at −80 °C until processing as described previously [26]. PCLS were homogenised at 4 °C using a Precellys homogeniser (Bertin Instruments, Basingstoke, UK) with 1 mm glass beads (Merck, Gillingham, UK); program: 2 cycles × 25 s at 5500 rpm with 5 s break. ATP was measured using ATP Bioluminescence Assay Kit HS II (Roche, Basel, Switzerland), and the protein content was measured using Protein Assay (Bio-Rad, Watford, UK).

### 4.5. Quantitative Real-Time PCR

The slices used for gene expression analysis were collected and processed with Allprotect tissue reagent (Qiagen, Manchester, UK) as per the manufacturer’s instruction. The RNA extraction was performed using an RNeasy Mini kit (Qiagen, Manchester, UK). To generate cDNA, 200 ng of RNA was used with the First Strand cDNA Synthesis Kit (ThermoFisher Scientific, Oxford, UK) according to the manufacturer’s protocol. Each sample was diluted 1:20 with RNAse-free water. Polymerase chain reaction was performed using SYBR green (ThermoFisher Scientific, Oxford, UK) in a 10 μL real-time PCR reaction. The following PCR conditions were used for all experiments: denaturation at 95 °C for 10 min, followed by 40 cycles at 95 °C for 15 s, then at 60 °C for 60 s. All primers were designed on Primer Express v3.0 and are available in Supplementary Table S1. Reactions were duplicated for each sample and quantified in the AriaMx Real-time PCR System (Agilent, Stockport, UK). Data were expressed as arbitrary units, and the relative expression of target genes (*FASN*, *SCD1*, *HMGCR*, *ELOVL6*) was corrected to the geometric average of two housekeeping genes (determined using BestKeeper tool, BK): *18S*, *GADPH*. Fold change across conditions was calculated by dividing the relative expression value of each gene by the geometric mean relative expression value from the control samples.

### 4.6. QuantiGene Gene Expression Analysis

Tissue samples treated with Allprotect tissue reagent (Qiagen, Manchester, UK) were processed with the QuantiGene Sample Processing Kit (ThermoFisher Scientific, Oxford, UK), and individual slices or baseline tissue was homogenised with a Precellys homogeniser (Bertin Instruments, Basingstoke, UK) at 4 °C in 600 µL of homogenising solution with 6 μL Proteinase K using CK14 ceramic beads (Bertin Instruments, Basingstoke, UK), program: 2 cycles × 15 s at 5000 rpm with 1 min break on ice. The gene expression analysis was performed using the QuantiGene Plex Assay (ThermoFisher Scientific, Oxford, UK) for the following genes: *SOX9*, *ACTA2*, *TIMP1*, *COL1A1*, *PDGFRB*, *RGS5*. The relative gene expression was calculated as the ratio of the median fluorescence intensity (MFI) of each target gene versus the geometric mean of the MFI of four housekeeping genes (*RPS29*, *HPRT1*, *RPLP0*, *TBP*). Then, each gene’s fold change was calculated by dividing the relative expression value of each sample by the geometric mean relative expression value from the control samples.

### 4.7. Analysis of Soluble Markers in Culture Supernatants

Culture supernatants were collected and centrifuged at 21,300× *g* for 10 min at 4 °C to remove cell debris and were stored at −80 °C until analysis. Cytokeratin-18 release in culture supernatant was used as a marker of epithelial cell death (i.e., hepatotoxicity). Both the M65 epitope (total cell death) and caspase-cleaved M30 epitope (apoptotic death) of cytokeratin-18 were measured in PCLS supernatants by ELISA (Peviva, Bioaxxes, Tewkesbury, UK). The procedure was performed as per the manufacturer’s instruction, and the total PCLS lysate was measured as maximal cytokeratin-18 release. Inflammation was assessed by measuring the concentration of TNFα, IL-6, IL-8 and IL-1β in the culture supernatant using a Luminex assay (Biotechne, Abingdon, UK) following manufacturer’s recommendation. Luminex data were acquired with a Luminex MagPix using xPonent 4.2 software (Luminex Corp., Austin, TX, USA), and quantitation data were generated using a 5-parameter logistic model for the standard curves. Undetectable data below the lowest standard curve concentrations were not considered as “missing data” but were assigned a half-minimum value for inclusion in statistical analyses, as previously published [7]. The secretion of TIMP-1 and TGF-β1 as an indication of profibrotic activity was measured using respective ELISA assays (Biotechne, Abingdon, UK). In order to quantify the release of the active form of TGF-β1, no acid activation was performed on analysed culture supernatants.

### 4.8. Statistical Analysis

The experimental design used in this study included patient-matched replicate PCLS cultures for all the conditions and all the timepoints. The intra-individual patient specificity implicit in this design was taken into consideration and “adjusted” for in our statistical models. If necessary, raw data were log-transformed to reduce skew. All the continuous variables measured in the study were compared using ANOVA models (or Mixed Effect when dealing with missing data). For significant models, individual multiple comparisons between treatments or conditions were then carried out with a Tukey’s post hoc multiple-comparison adjustment. When time durations were not relevant to the analysis, conditions and experimental groups were only compared within each timepoint. This is indicated in the respective graphs. All the analyses were performed using GraphPad Prism (9.4.1). All the tests were performed at a two-tailed 5% significance level (alpha = 0.05). The statistical significance is indicated on the graphs as follows: * *p* ≤ 0.05, ** *p* ≤ 0.01, *** *p* ≤ 0.001, **** *p* ≤ 0.0001.

## 5. Conclusions

The current study describes the critical properties of human PCLS in modelling ALD in vitro, focusing on multiple molecular processes associated with disease pathogenesis. We show that features central to the progression of ALD are reflected in the PCLS model, including upregulation of lipid synthesis, hepatoxicity, inflammation and fibrogenesis. Our analysis considers essential characteristics intrinsic to the PCLS model (i.e., cut effect) and their impact on ALD modelling. We demonstrate that to replicate more reliably pathophysiological events observed in patients, such as bacterial translocation, the culture conditions of the slices can be efficiently modulated, confirming the versatility of this model for ALD studies and the potential of using it for the screening of therapies which synergistically target multiple pathways.

## Figures and Tables

**Table 1:** Baseline characteristics of liver tissue donors for precision-cut liver slices preparation.

	Demographics				Background Liver		Tumour	Treatment (Y/N)	Alcohol	
SUBJECT ID	Sex	Age	Ethnicity	BMI	Fibrosis Score	Other Observations	Aetiology		Current/Former	Units/Week
PCLS-130-KCH	F	81	Caucasian	28.97	F0–F1 *	Areas of parenchyma atrophy and F1 with mild CL PSF. 10% steatosis	CRLM	Y	N	UA
PCLS-132-KCH	M	39	Caucasian	UA	F0–F3 *	Areas of F2–3, secondary to SOS with marked PSF and NRH20% steatosis	CRLM	N	UA	UA
PCLS-149-KCH	F	37	Caucasian	19.36	F0	Rare pp delicate fibrous spurs	CRLM	Y	UA	UA
PCLS-156-KCH	F	69	Caucasian	17.3	F0 *	Very focal pp delicate expansion and focal CL PSF	CRLM	Y	current	<14
PCLS-159-KCH	M	40	Asian	24.8	F0	<5% steatosis	CRLM	N	N	UA
PCLS-190-KCH	M	60	Caucasian	26.7	F0	30% steatosis	CRLM	N	N	UA
PCLS-215-KCH	M	50	Caucasian	28.7	F0–F1	Variable pp delicate fibrosis and patchy CL PSF (mild SOS)	CRLM	N	UA	UA

Abbreviations: BMI—body mass index; UA—unavailable; CLRM—colorectal liver metastasis. Fibrosis scoring: F0—no fibrosis; F1—portal fibrosis; F2—periportal fibrosis extension; F3—bridging; * patchy fibrotic areas; CL—centrilobular; PSF—perisinusoidal fibrosis; SOS—sinusoidal obstruction syndrome; NRH—nodular regenerative hyperplasia; pp—periportal.

## Data Availability

The data presented in this study are contained within the article and raw data are available on request from the corresponding author.

## Supplementary Materials

The supporting information can be downloaded at: https://www.mdpi.com/article/10.3390/ijms25010150/s1.
