# Supplementary material for: Human Precision-Cut Liver Slices: A Potential Platform to Study Alcohol-Related Liver Disease
Source: Int J Mol Sci. 2023 Dec 21;25(1):150. doi: 10.3390/ijms25010150 (PMC10778645; doi:10.3390/ijms25010150)
Supplement: Supplementary file 1 [file ijms-25-00150-s001.zip › ijms-2541604-supplementary.pdf]

Supplementary figures

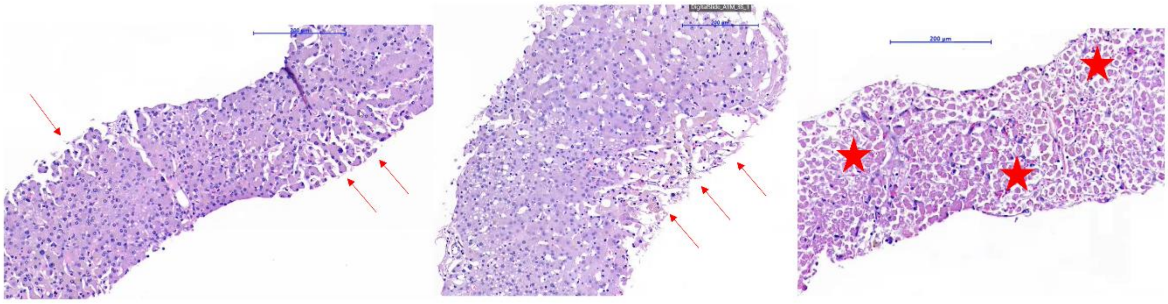

Before recovery After recovery Day 3

Supplementary figure S1. Representative H&E staining of non-viable PCLS culture. This culture failed the assessment of viability and was not considered in the overall results. Autolytic changes (red arrows) can be seen immediately following PCLS preparation (Before recovery and After recovery). Areas of necrosis (red stars) in the cultured slice (Day 3).

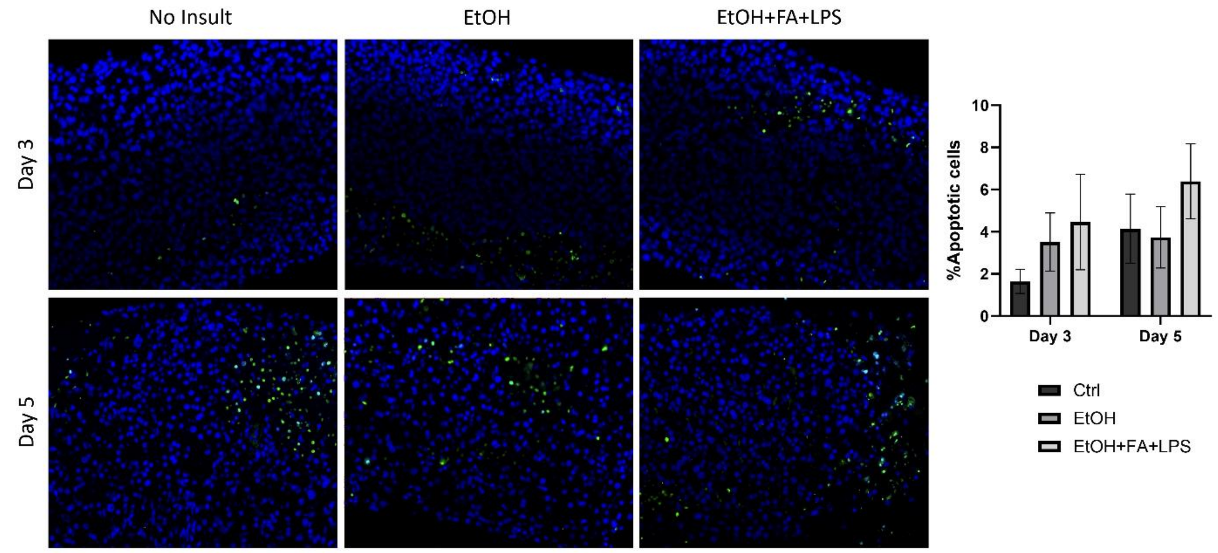

Supplementary figure S2. TUNEL staining of PCLS indicating apoptotic cells in PCLS treated with alcohol insults for 3 and 5 days. Staining for apoptotic cells was done using DeadEnd™ Fluorometric TUNEL System Kit (Promega) following the manufacturer's recommendations. Magnification 400x. Mean ± SD. 3 images per condition. n(patients)=1.

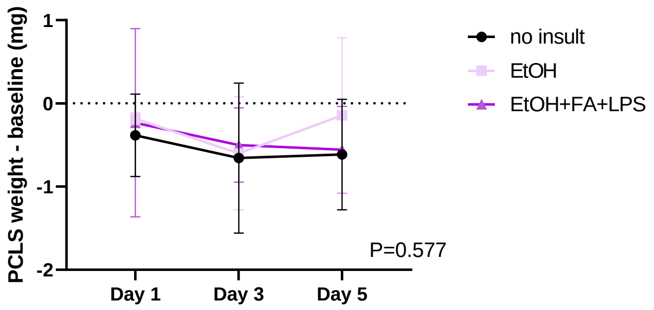

Supplementary figure S3. Change in PCLS weight over time. Mean ± SD. n(patients)=2, 2-5 samples per patient.

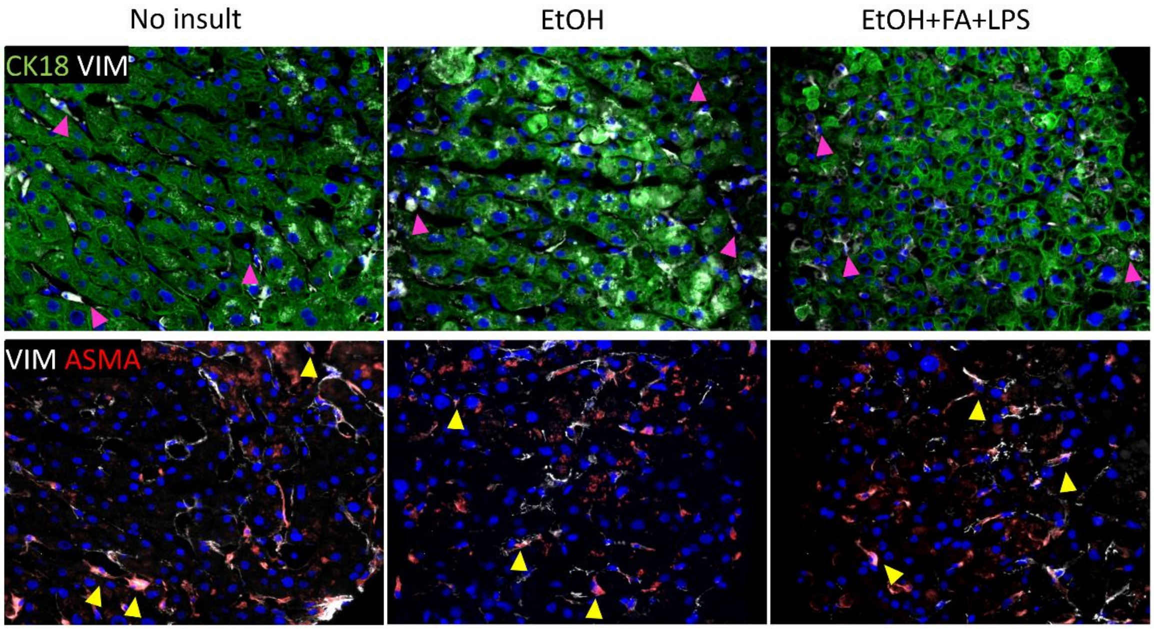

Supplementary figure S4. PCLS maintain hepatocytes (cytokeratin-18+, Dako M7010) and mesenchymal cells (vimentin+, R&D MAB2105) in slices treated with alcohol insults (Day 3). Magnification 400x, pink arrows: mesenchymal cells, yellow arrows: a-smooth muscle actin (ThermoFisher Scientific AB_2572996) and vimentin positive cells indicative of activated hepatic stellate cells in the slices.

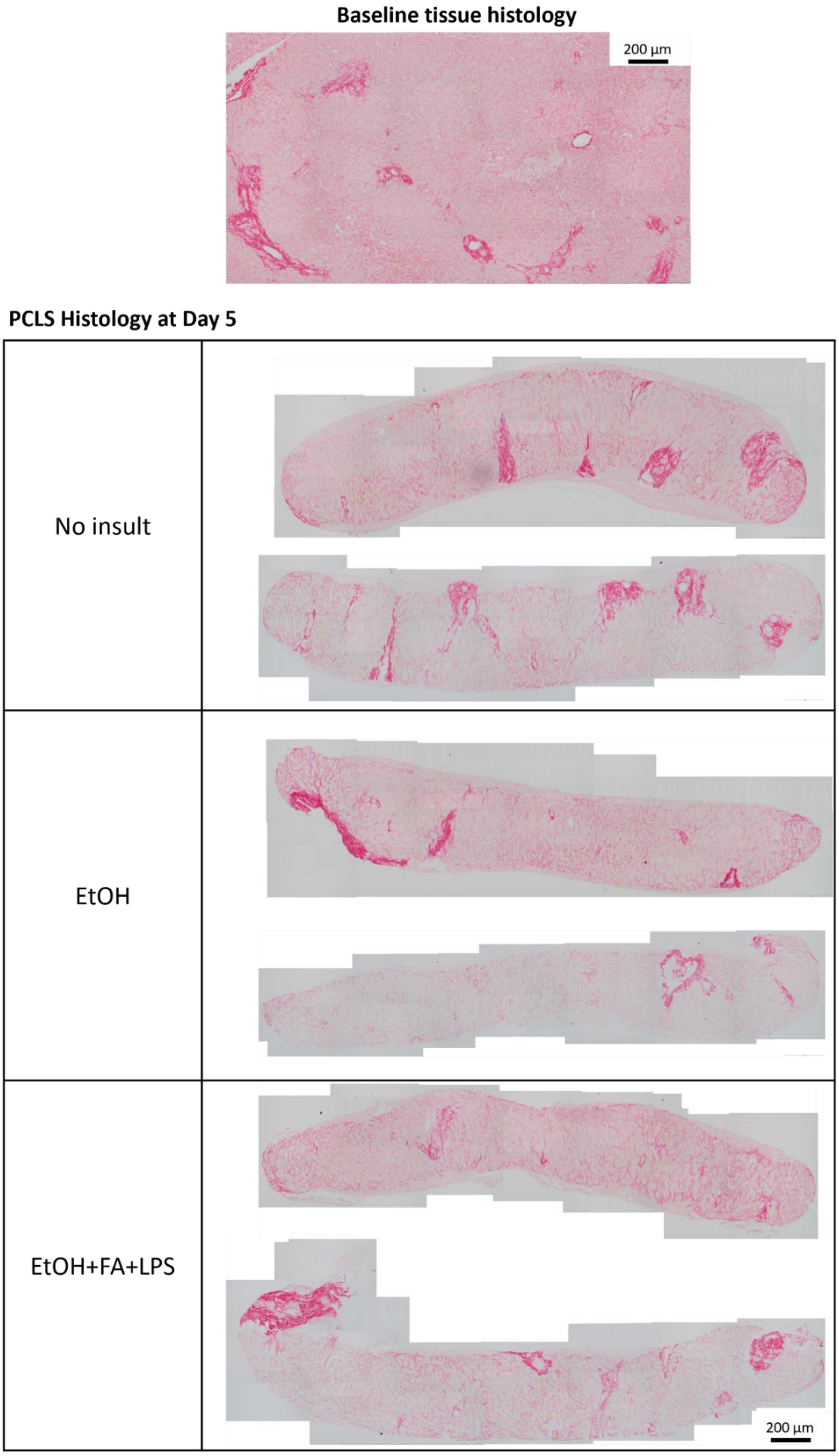

Supplementary figure S5. Picrosirius red staining of the PCLS exposed to alcohol insults for 5 days compared to the baseline tissue used for PCLS preparation.

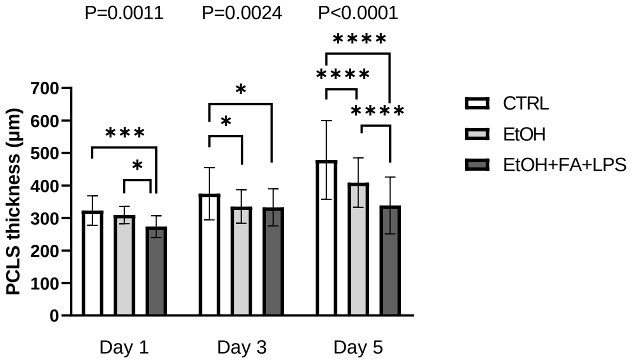

Supplementary figure S6. Change in PCLS thickness over time. Mean ± SD. n(patients)=3-7 per timepoint, 1-4 samples per patient.

* P ≤ 0.05, ** P ≤ 0.01, *** P ≤ 0.001, **** P ≤ 0.0001

Supplementary table S1. Primers sequence for the Quantitative real-time PCR.

Gene	Forward	Reverse
HMGCR	TGATTGACCTTTCCAGAGCAAG	CTAAAATTGCCATTCCACGAGC
FASN	GCAAATTCGACCTTCTCCAGAA	GTAGGACCCCGTGGAATGTC
ELOVL6	AACGAGCAAAGTTTGAACTGAGG	TCGAAGAGCACCGAATATACTGA
SCD1	TCTAGCTCCTATACCACCACCA	TCGTCTCCAACTTATCTCCTCC
18S	CGGCTACCACATCCAAGGAA	GCTGGAATTACCGCGGCT
GADPH	CATGGCCTCCAAGGAGTAAG	AGGGGTCTACATGGCAACTG
